# Strategies for improving recruitment of pregnant women to clinical research: An evaluation of social media versus traditional offline methods

**DOI:** 10.1177/20552076221095707

**Published:** 2022-05-03

**Authors:** Kelsey M Cochrane, Jennifer A Hutcheon, Crystal D Karakochuk

**Affiliations:** 1 Department of Food, Nutrition and Health, Faculty of Land and Food Systems, 8166 The University of British Columbia, Vancouver, BC, Canada; 2 512469 BC Children’s Hospital Research Institute, Vancouver, BC, Canada; 3 Department of Obstetrics and Gynaecology, Faculty of Medicine, 8166 The University of British Columbia, Vancouver, BC, Canada

**Keywords:** Pregnancy, clinical trials, social media, Facebook, womens health

## Abstract

**Objectives:**

To evaluate the recruitment of pregnant women for a clinical trial in Vancouver, Canada, via social media versus offline methods and to explore optimization of social media campaigns.

**Methods:**

Facebook was used to run nine social media campaigns (15 weeks total, CA$675). Offline methods were used concurrently over 64 weeks (printing costs: CA$300). The cost, rate of recruitment and conversion rate in each group was calculated. Performance metrics of social media campaigns (reach, impressions, clicks, inquiries, enrolments) were recorded. Linear regression was used to explore the association between metrics and dollars spent per campaign.

**Results:**

In total, n = 481 inquiries were received: n = 51 (11%) via offline methods and n = 430 (89%) via social media. Enrolees (n = 60 total) included n = 24 (40%) and n = 36 (60%) via offline and social media methods, respectively. Gestational weeks upon inquiry (n = 251; mean ± SD) were not different among groups (offline: 13.3 ± 4.7; social media: 13.2 ± 5.6). Direct cost per enrolee was CA$13 and CA$19 via offline and social media methods, respectively (however, this does not include cost of labour). The rate of recruitment was approximately six times faster via social media. However, the conversion rate was higher via offline methods than social media (47% vs. 8%). The amount spent per campaign was significantly associated with improved clicks and inquiries, but not enrolments.

**Conclusions:**

Social media was more efficient and effective than offline methods. We gained numerous insights for optimization of social media campaigns (dollars spent, attribution setting, photo testing, automatic optimization) to increase clicks and inquiries, however, this does not necessarily increase enrolments, which was more dependent on study-specific factors (e.g. time of year, study design).

## Introduction

Participant recruitment is considered one of the greatest obstacles in conducting clinical research and has been reported as the most significant contributor to randomized trial delays.^1,2^ It has been estimated that nearly 80% of pharmaceutical drug trials in the United States fail to meet recruitment timelines, and in a review of 114 clinical trials in the United Kingdom, only 31% met their original recruitment goal.^3,4^ In addition to increased costs and potential loss of funding, inadequate recruitment may cause reduction in data quality, poor demographic diversity and generalizability, underpowered analyses and ultimately, failure to complete the trial.^5^ Strong recruitment planning, which is tailored for a specific population and utilizes evidence-based strategies, can greatly increase the likelihood of meeting enrolment targets.^2,5^

There are numerous unique challenges in recruiting pregnant women.^6,7^ Medical clinics are often the primary recruitment site, with strategies including physician referrals and distributing printed materials such as posters or brochures.^7–9^ These strategies (referred to hereinafter as ‘offline’ methods) are labour-intensive and require established rapport between research and clinic personnel. Additionally, it can be difficult to target women at the appropriate gestational age, particularly those in very early pregnancy, as first prenatal appointments typically occur at 8–12 weeks gestation. Participant diversity is inherently limited by the clinic location and to those within relatively close proximity.^10,11^ As such, perinatal research often has poor geographical and demographic diversity, and those in earlier pregnancy are often underrepresented.^7,12^

Social media encompasses various websites and applications (e.g. Facebook, Instagram, Twitter), which allow users to create and share information and participate in social networking.^13^ Use of these platforms has grown tremendously in recent years, with 1.9 billion global daily Facebook users reported in 2021.^14^ This growth was exacerbated with onset of the global COVID-19 pandemic and a requirement to shift activities online (a change hypothesized to persist post-COVID-19).^15,16^ Thus, use of social media for participant recruitment is an increasingly viable and attractive option. In a 2020 systematic review and meta-analysis of 61 studies, social media was significantly more efficient than offline methods for participant recruitment (incidence rate ratio = 4.17, 95% CI 1.12–15.59).^2^ Shorter term (generally <30 days), intermittent campaigns appeared to be more successful, but optimal timing is not well described.^2^ However, offline recruitment had a significantly higher conversion rate (percentage of screened participants who ultimately enrolled).^2^ A 2021 systematic review found that social media reached more participants than offline methods, but only ∼20% of those who inquired via social media enrolled.^17^ Cost estimates vary greatly by individual studies, but both reviews reported that recruitment via social media is generally more cost effective than use of offline methods.^2,17^

Facebook's advertising platform allows researchers to create a customized advertisement, targeted to reach a specific population, while specifying the total dollars spent (lifetime budget) over a select period of time (referred to as a ‘campaign’). An objective is specified for each campaign, with options that include: ‘reach’ (aims to show the advertisement to the maximum number of people), ‘messages’ (encourages people to interact with you via messaging), ‘engagement’ (increases the number of likes, page comments, etc.) and others. Placement of advertisements can be divided between various Facebook platforms, including Facebook and Instagram. ‘Campaign budget optimization’ and ‘automatic placement’ can be used to monitor the lifetime budget of a campaign and allocate the placement of advertisements to achieve the best results based on the specified objective.

Use of social media for recruitment of pregnant women in various types of research (clinical trials, surveys and epidemiological studies) has been explored.^12,18–24^ In two of these studies, pregnant women were recruited for Canadian-based clinical trials.^12,18^ First, Shere et al. posted study information for free on various social media platforms (Facebook, Twitter) and found a 12-fold increase in recruitment rate as compared to offline methods.^12^ Alternatively, Adam et al. ran paid Facebook campaigns (26 non-consecutive days), while concurrently using offline methods over 7 months.^18^ Facebook was more cost effective and efficient (rate of recruitment approximately nine times greater), and recruited women earlier in their pregnancy, than offline methods.^18^ Whether demographic characteristics of participants reached varies by recruitment method is unclear. Group differences by recruitment strategy is an important consideration, as this could bias results.^25^ However, improving diversity in research would improve generalizability of findings. Conflicting reports on how social media affects participant diversity have been published.^17,19,25^

We enrolled n = 60 healthy pregnant women in a clinical trial in Vancouver, Canada, using a series of paid Facebook campaigns and traditional offline methods.^26^ The aims of this study were twofold: (1) to evaluate and compare results of recruitment via social media and offline methods (including demographic characteristics, rate of recruitment and direct costs); and (2) to explore optimization of intermittent online campaigns (including dollars spent, campaign duration, photo used and settings in Facebook ad design (e.g. attribution setting)).

## Methods

### Study population

Full study details are published elsewhere,^26^ but in brief, n = 60 pregnant women were enrolled to a proof-of-concept pilot study of supplementation with folic acid or (6*S*)-5-methyltetrahydrofolic acid for 16 weeks of pregnancy, starting at 8–21 weeks gestation. The study was randomized and double-blinded. See Supplemental Table 1 for Consolidated Standards of Reporting Trials (CONSORT) checklist. Inclusion criteria included: singleton pregnancy, living in Vancouver, Canada, ≤21 weeks’ gestation and 19–42 years of age. Exclusion criteria included: having a medical condition, currently taking medications or lifestyle factors (current smoking, alcohol consumption, recreational drug use, a pre-pregnancy body mass index (BMI) ≥30 kg/m^2^) which may interfere with B-vitamin metabolism,^27^ and those medium to high risk for development of a neural tube defect-effected pregnancy.^27^

### Social media recruitment

A Facebook account was created for the ‘UBC Folate in Pregnancy Study’ to run a series of nine campaigns throughout October 2019 to January 2021. Campaign timelines ranged from 10 to 18 days with various lifetime budgets (CA$50, CA$75 and CA$100). Overall, campaigns ran for 103 days (∼15 weeks) and CA$675 was spent. Campaigns were targeted to reach women aged 19–42 years, living in Vancouver, Canada (within a 25-mile radius), who match the following interests: pregnancy, nutrition and pregnancy, prenatal nutrition, motherhood and fit pregnancy. Advertisements were automatically optimized by Facebook using campaign budget optimization and automatic placement. The default attribution setting was used for all campaigns. Attribution setting is a defined period of time in which interactions with the advertisement (clicks or views) are used to inform campaign optimization. For the first eight campaigns, this setting was 28-day click, 1-day view. In January 2021, Facebook's default setting changed to 7-day click, 1-day view, so this was used for the final (‘9th’) campaign. The objective chosen for all campaigns was ‘messages’. Those who saw the ad were prompted to click ‘send message’, which would automatically connect them to study staff via Facebook Messenger. Messages were monitored by the study's research coordinator, who provided study information, fielded questions, assessed eligibility and enrolled participants.

**Figure 1.**
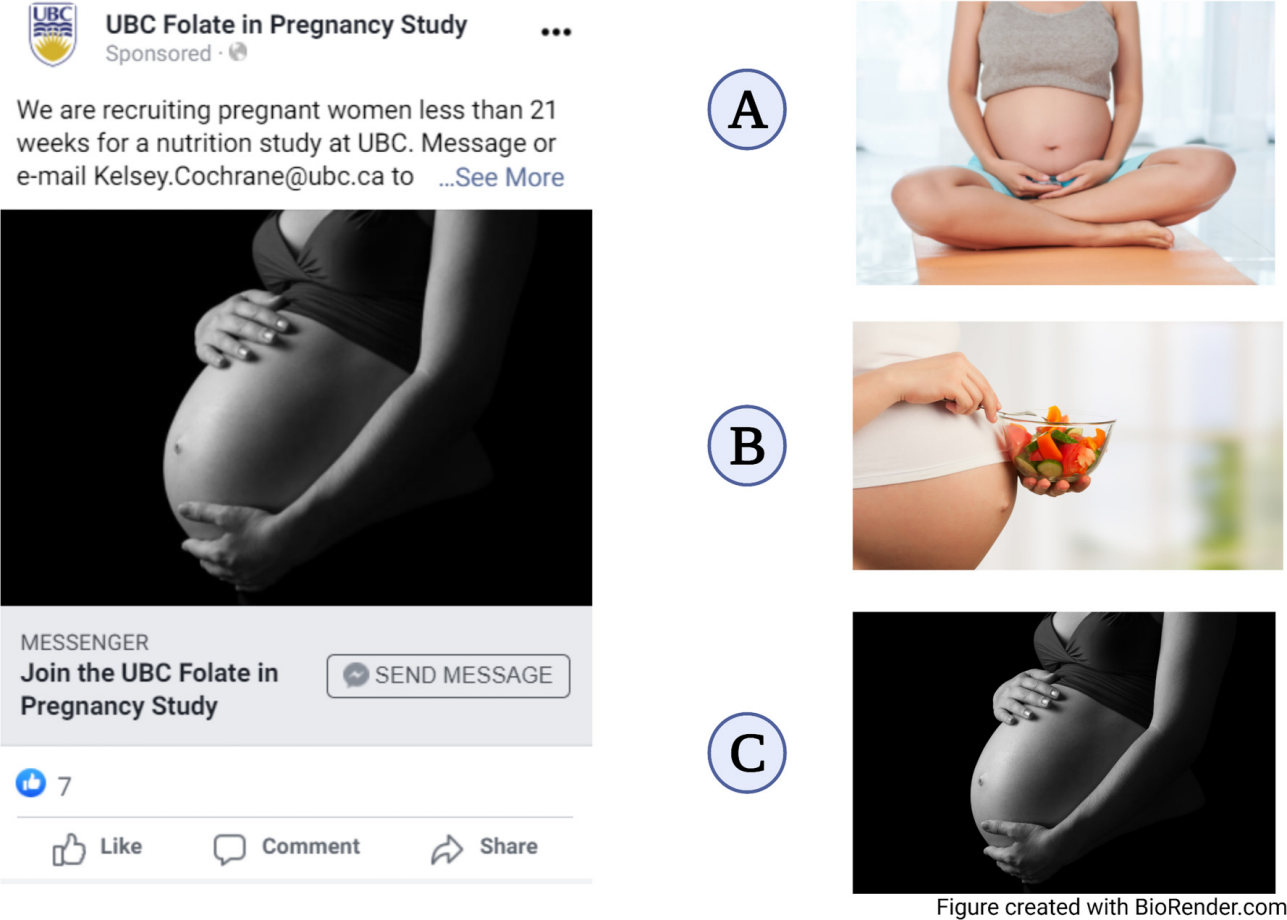
Social media campaigns and photos ‘A’, ‘B’ and ‘C’.

The primary text in the advertisement indicated current recruitment for pregnant women less than 21 weeks gestation for a nutrition study at UBC. Ad text was approved by Facebook advertising prior to going live, meeting requirements of the privacy policy (e.g. to ensure that personal attributes, such as pregnancy state, are not directly inferred). Participants could inquire using the research coordinator's contact information (displayed in the ad) or by clicking ‘send message’. See Figure 1(a) for an example of social media advertisements. Three different photos (Figure 1(b): ‘A’, ‘B’ and ‘C’) were subbed into the advertisement throughout campaigns (purchased at Can Stock Photo). Photos similar to those used by Adam et al. were selected, as they recruited pregnant individuals for a similar Canadian clinical nutrition trial.^18^ Photos were evaluated with A/B testing; this allows two options for a variable in a campaign (e.g. photo) to run simultaneously to determine which performs best. When running an A/B test, the total budget is allocated 50/50 to each ‘version’ of the advertisement (e.g. for a $100 lifetime budget, $50 is allocated to advertisements with each photo). Photos featured in each campaign were as follows: campaigns ‘1-5’, photo ‘A’; campaign ‘6’, photo ‘B’; campaign ‘7’, A/B test of photos ‘A’ and ‘B’; campaign ‘8’, A/B test of photos ‘A’ and ‘C’; campaign ‘9’, photo ‘C’.

### Offline recruitment

Various offline recruitment methods were used throughout September 2019 to December 2020 (16 months or ∼64 weeks total). The predominant strategy included distribution of posters (which featured Photo ‘A’) across several locations in Vancouver, including the University of British Columbia (UBC), the BC Children's and Women's Hospital and Research Institute, medical and prenatal clinics and various retail and community establishments for pregnant women (prenatal classes, fitness studios, maternity clothing stores). Posters were distributed monthly and refreshed as needed across ∼50 different locations from September 2019 to March 2020. Overall, 350 posters (CA$300 printing costs) were distributed. Upon onset of COVID-19 (April 2020 to December 2020), study information was shared electronically with accepting medical clinics and community establishments. Finally, study details were presented at the BC Women's Hospital Obstetrics & Gynaecology Grand Rounds and the Midwife Grand Rounds in November 2019 and September 2020, respectively, to inform clinicians about the study and how to refer interested patients.

### Statistical analyses

#### Aim 1

Descriptive statistics (mean ± SD, median and IQR, or counts with percentages) were used to present characteristics of enrolled participants and gestational weeks (as available) of those who inquired. Differences between groups were assessed using a two-sample *t*-test (for continuous, normally distributed variables) or Pearson chi-square test (for categorial variables). Fisher's exact test was used for categorical variables when cell frequencies were <5. Direct cost (not including labour) per enrolled participant was determined by dividing the total cost of each method by the number of participants enrolled. Rate of recruitment (inquiries/week and enrolments/week) for each recruitment strategy was determined by dividing the total number of participants who inquired and enrolled by the total weeks. The conversion rate for each method (enrolments/inquiries) was calculated to determine the number of inquiries who enrolled. Odds of enrolment after inquiring in each group was evaluated with an odds ratio (OR) and 95% CI.

#### Aim 2

The following metrics were recorded for each social media campaign: reach (number of different people who saw the ad), impressions (the number of times the ad was on a screen; unlike reach, this may include multiple views by the same person), clicks, cost per click, inquiries (sent within Messenger and via e-mail), cost per inquiry, enrolments and cost per enrolment. Reach, impressions and clicks were available via Facebook analytics, while inquiries and enrolments were recorded by the research coordinator. Linear regression was used to explore the association between campaign metrics, including association of lifetime budget per campaign (controlling for campaign duration) with clicks, inquiries and enrolments, and association of clicks and inquiries with enrolments. A/B photo tests were evaluated using metrics available via Facebook analytics (reach, impressions, clicks). *P*-values <0.05 were considered statistically significant. Statistical analyses were performed using Stata 16.1 (Stata Corp, Texas, USA).

## Results

### Aim 1: comparison of recruitment via social media versus offline methods

**Figure 2.**
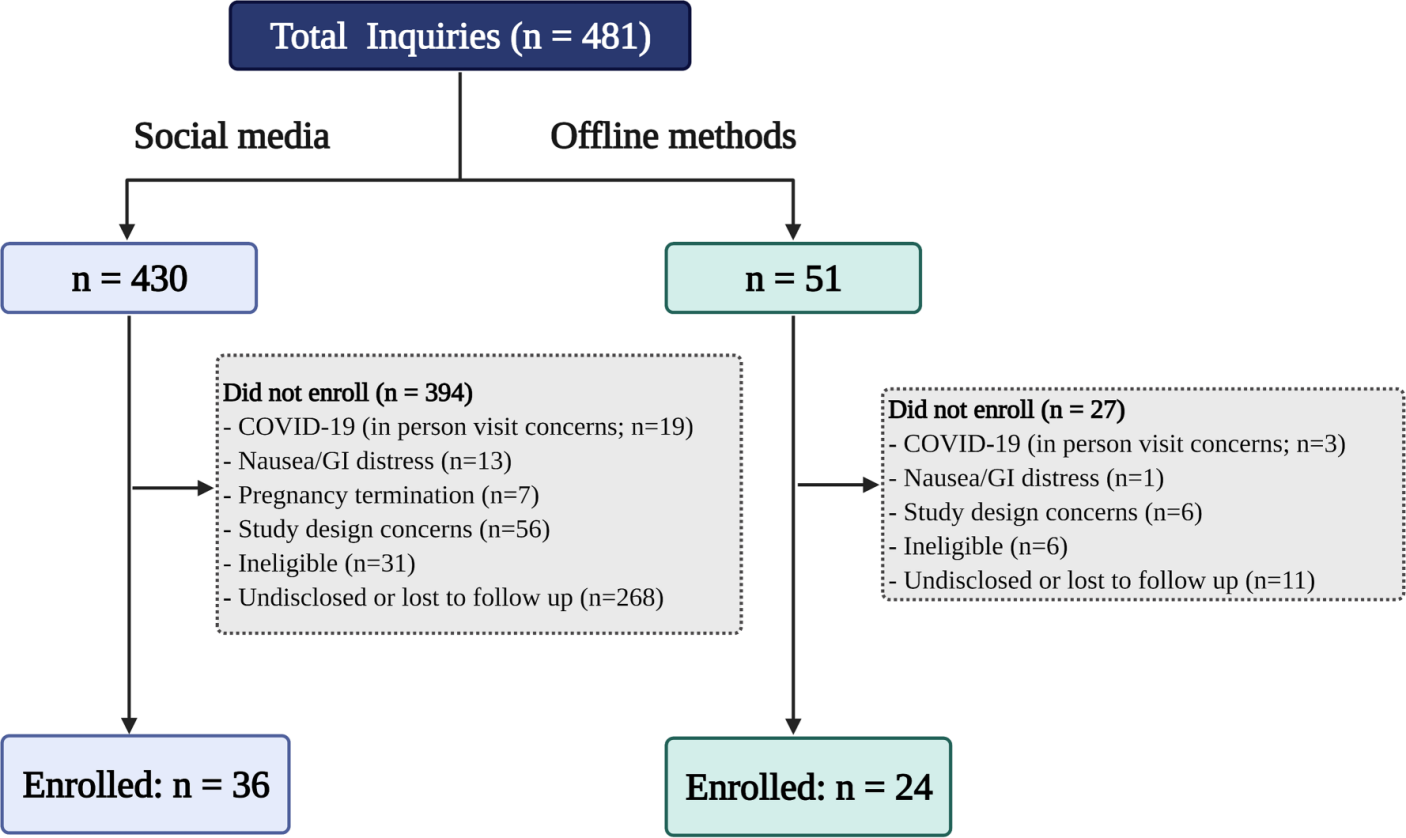
Participant flow chart.

In total, n = 481 inquiries were received from September 2019 to January 2021 to participate in the clinical trial. See Figure 2 for participant flow chart. Briefly, n = 430 (89%) inquired after seeing a social media ad and n = 51 (11%) inquiries were a result of offline methods.

Gestational weeks were reported by n = 251 (52%) women upon inquiry. Of these, n = 208 (83%) inquired via social media and n = 43 (17%) via offline methods. Mean ± SD gestational weeks at inquiry was comparable between the social media and offline groups: 13.2 ± 5.6 and 13.3 ± 4.7 weeks gestation, respectively (*P* = .96). Demographic characteristics of enrolled participants (n = 60) are presented in Table 1. Briefly, n = 36 (60%) and n = 24 (40%) enrolled following inquiries from social media and offline methods, respectively. For offline methods, n = 15 (63%) inquired after seeing a study poster and n = 9 (37%) inquired following other offline strategies (clinician recommendation, word of mouth). Although not statistically significant, a higher proportion of those recruited via offline methods had University-level education and an annual household income of >CA$100,000 per year (88% and 58%, respectively), as compared to those recruited via social media (78% and 47%, respectively).

**Table 1.** Demographic characteristics of enrolled participants by recruitment strategy.

	Recruitment method		
Characteristic	Offline	Social media	*P* value
Number of participants (n, %)	24 (40)	36 (60)	
Age, mean (SD)	33 (3.2)	33 (3.6)	0.67
**Ethnicity (n, %)**			0.99
European	14 (58)	20 (56)	
South, East & Southeast Asian	5 (21)	8 (22)	
Hispanic/Latino	3 (13)	4 (11)	
Middle Eastern	1 (4)	1 (3)	
Biracial	1 (4)	3 (8)	
**Education (n, %)**			0.82
Highschool	0 (0)	2 (5)	
College	3 (12)	6 (17)	
Undergraduate	11 (46)	14 (39)	
Graduate	10 (42)	14 (39)	
**Household income (CA$)/year (n, %)**			0.23
<20,000	0 (0)	1 (3)	
20,000–50,000	4 (17)	2 (6)	
50,000–100,000	6 (25)	15 (44)	
>100,000	14 (58)	16 (47)	
Pre-pregnancy BMI, mean (SD)	22.2 (2.6)	23.4 (2.8)	0.11
Weeks gestation at inquiry, mean (SD)	13.5 (4.7)	13.0 (5.0)	0.72
**Parity (n, %)**			0.81
Nulliparous	18 (75)	26 (72)	
Multiparous	6 (25)	10 (28)	

Note: Age, pre-pregnancy BMI and weeks gestation at inquiry were compared with a two-sample *t*-test. Ethnicity, education and household income were compared with a Fisher's exact test. Parity was compared with a Pearson chi-square test.

Direct cost per enrolled participant was CA$19 (CA$675 / n = 36) in those recruited via social media and CA$13 (CA$300 / n = 24) in those recruited via offline methods. The overall rate of inquiries per week was 28.7 (n = 430/15 weeks) using social media and 0.80 (n = 51/64 weeks) using offline methods. The overall rate of enrolments per week was 2.4 (n = 36/15 weeks) using social media and 0.38 (n = 24/64 weeks) using offline methods. The conversion rate (inquiries which enrolled) was 8% in the social media group (n = 36/n = 430) and 47% in the offline methods group (n = 24/n = 51). The odds of enrolment via offline methods was 5.6 times higher than after seeing an online ad (OR = 5.6, 95% CI 2.9–10.5).

### Aim 2: explore optimization of social media campaigns

**Figure 3.**
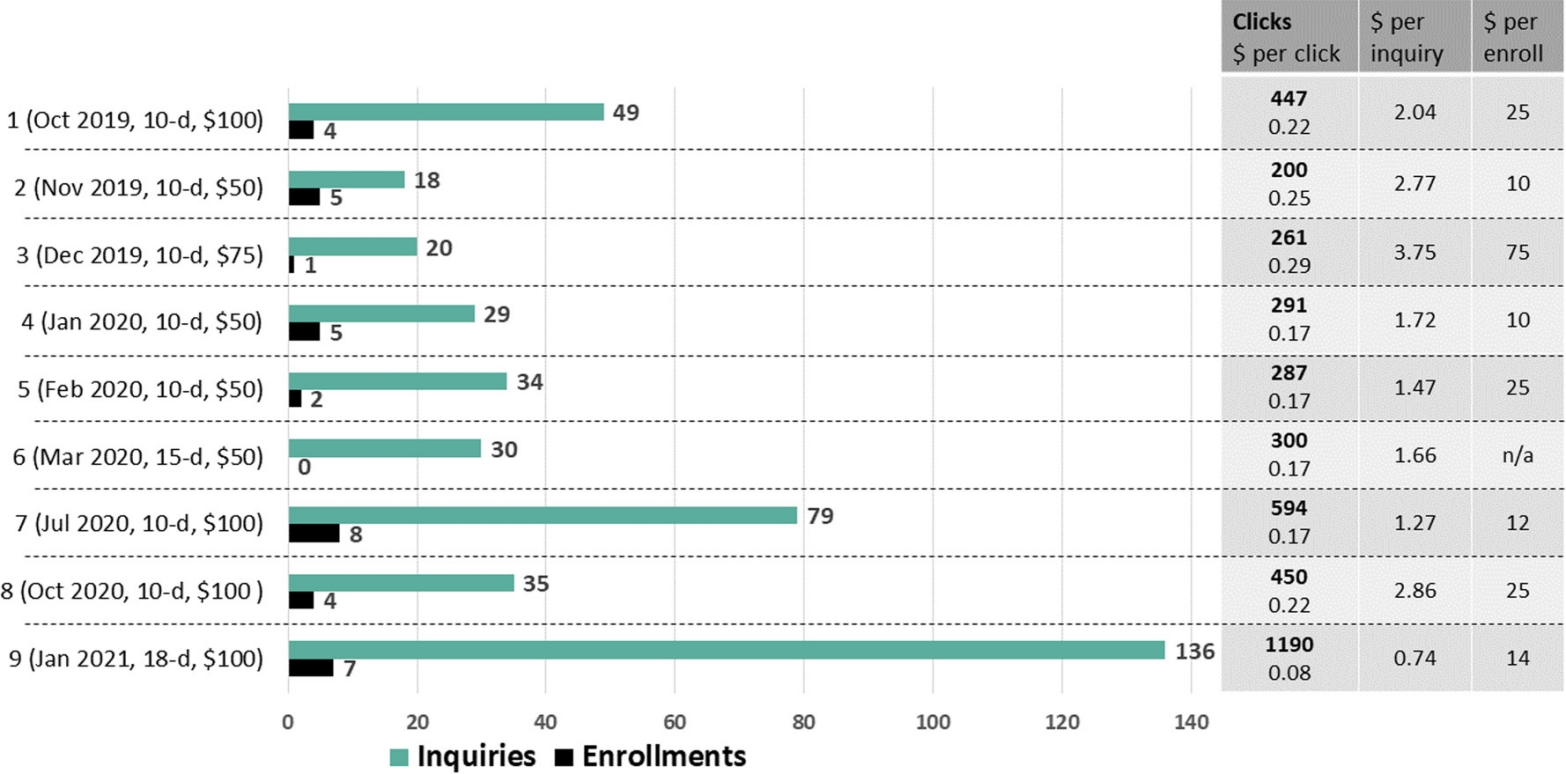
Summary of clicks, inquiries and enrolments per campaign and cost analysis by each metric (dollar amounts in CA$).

A full summary of metrics from the social media campaigns can be found in Supplemental Table 2. Briefly, campaigns reached a median of 2970 women (IQR 2872–4025), made a median of 6130 (IQR 4951–7547) impressions and resulted in a median of 300 clicks (IQR 287–450). Impressions, reach and clicks are inherently correlated (*P *< 0.001), as impressions include all women who were reached, but also captures the number of times the advertisement was on a screen, and clicks are recorded following a click of the advertisement. Total clicks, inquiries and enrolments per campaign, and the cost analysis of each metric, are presented in Figure 3. Lifetime budget (CA$50 to CA$100), controlling for campaign duration (10–18 days), was positively associated with the number of clicks (*β* = 7.01, 95% CI 2.25–11.77; *P* = .01) and inquiries (β = .82, 95% CI.08–1.5; *P* = 0.04), but not the number of enrolments (*P* = 0.19). Although increased clicks (*P* =  0.09) and inquiries (*P* = 0.06) tended to result in increased enrolments, the associations did not reach significance.

Metrics for photos ‘A’ and ‘B’ in the first A/B test (campaign ‘7’, July 2020) were: reach 3010 and 3072; impressions 5550 and 4891; clicks 314 and 280. Photo ‘A’ was more successful, with Facebook analytics predicting a 92% chance of achieving the same result upon test repeat. In the second A/B test (campaign ‘8’, October 2020), metrics for photos ‘A’ and ‘C’ were: reach 1542 and 1647; impressions 3305 and 3408; clicks 203 and 247. Photo ‘C’ was more successful, with Facebook analytics predicting a 68% chance of achieving the same result upon test repeat.

## Discussion

Our findings support that using social media improves the efficiency (speed of recruitment) and overall effectiveness (number of participants enrolled) of recruitment compared to traditional offline methods. Additionally, we provide novel data regarding the demographic characteristics of pregnant women recruited for clinical research via social media compared to offline methods and strategies for optimizing online campaigns.

Contrary to previous findings, we did not find a significant difference in gestational weeks among pregnant women recruited via social media versus offline methods. Adam et al. recruited pregnant women (n = 70) for a similar clinical trial, and reported that those recruited via social media (n = 25) were significantly earlier in their pregnancy than those recruited via offline methods (n = 45), finding a mean ± SD gestational weeks of 12.6 ± 3.7 and 14.7 ± 3.8 in social media and offline methods groups, respectively.^18^ We examined gestational age in a much larger cohort (n = 251) and did not find a difference between groups (13.2 ± 5.6 and 13.3 ± 4.7 via social media and offline methods, respectively). In agreement with Adam et al., it does appear that most women inquire for participation early in their first trimester (once >12 weeks gestation). National survey data in the United States among n = 11,928 pregnant women reported that the mean gestational age of pregnancy awareness is 5.5 weeks.^28^ Exact reasons why women tended to wait until >12 weeks to inquire is unknown; however, it is unsurprising. As previously stated, first prenatal ultrasound appointments to confirm foetal viability typically occur at 8–12 weeks gestation, thus, it is reasonable that women would not inquire about pregnancy-related clinical research until after this time. This data provides evidence for feasible gestational age ranges for future research.

We did not find significant differences in ethnicity between women enrolled via social media (n = 36) versus online methods (n = 24). This is contrary to findings from Admon et al., who recruited a more racially diverse group of pregnant women based in the United States for an online survey using social media (n = 759) as compared to clinic-based recruitment (n = 190).^19^ Differences in study design (recruitment for a clinical trial vs online survey) may have contributed to these findings. Overall, we note that members of a visible minority were moderately under-represented in our study. According to 2016 Canadian Census data, 52% of Vancouver's population are members of a visible minority (predominantly of South, East and Southeast Asian ancestry);^29^ 43% (n = 26) of participants in the current study identified as a visible minority. Although we selected photos showing only a pregnant abdomen, we note that the lack of racial diversity in the photos may have contributed to this. Future studies should include photos with diverse skin tones.

In agreement with Admon et al., we found that women recruited via offline methods tended to have higher levels of University-level education and reported a higher annual household income (>CA$100,000 per year) than those recruited via social media. However, these findings were not statistically significant. Placement of study posters around the UBC campus and the BC Children's and Women's Hospital Research Institute (where study visits took place) may explain these findings, as many with University-level education work and study at these locations. These are important findings, as universities and research institutes are often where clinical trials take place, thus, are a common location for poster distribution. As per 2016 Canadian Census data, 36% of females (ages 25–64) in Vancouver, Canada, have University-level education, and 20% have an annual household income below the national poverty line (∼CA$40,000/year for a couple with one child).^29,30^ Thus, although diversity (as per education and income) was improved in those recruited via social media, overall, participants in both groups were above provincial averages. It is possible that social media did reach a more diverse range of individuals, but the effect was diluted due to the location of study visits and bias towards those who live and work nearby. Expanding geographical reach of offline methods, including recruitment at various non-profit organizations (e.g. food banks, community centres) and medical centres in lower-income neighbourhoods, may be effective strategies to increase the reach of those with lower socioeconomic status.^31^ Additionally, clinical researchers should offer study visits at a variety of locations whenever feasible.

Ultimately, social media was more effective than offline recruitment, resulting in n = 36 versus n = 24 participants enrolled, respectively. There was also a stark difference in efficiency, as the participants were recruited approximately six times faster via social media than offline methods. This is similar to previous findings.^2,18^ A critical and unique consideration in this study is the effect of COVID-19. Offline strategies were shifted from manual distribution of study materials throughout the community (September 2019 to March 2020) to sharing them electronically (April 2020 to December 2020). Enrolments per month decreased from approximately two per month to one per month upon shifting offline strategies after March 2020. A factor to consider for the efficiency of social media recruitment is that although social media ads were active for only 15 weeks, they spanned 64 weeks in total (October 2019–January 2021). However, inquiries and enrolments only occurred during active social media campaigns phases. Regardless, it is unknown if we would have obtained the same rate of recruitment via social media if the 15 weeks were run continuously. Intermittent campaigns have been reported as more effective than continuous ones,^2^ possibly due to minimizing saturation of the target population, and in our case, access to a ‘new wave’ of women becoming pregnant. Spacing of our online campaigns was affected by COVID-19, as spacing increased from one per month to every 3–4 months after March 2020. These differences in spacing did not appear to alter campaign performance. Overall, optimal spacing between campaigns requires further research.

Cost estimates require careful interpretation. Although the direct cost per enrolled participant was CA$19 and CA$13 via social media and offline methods, respectively, costs in the offline groups only contributed to those recruited via posters (n = 15), while the remaining participants in this group (n = 9) were recruited following other offline strategies. Thus, estimation of direct costs per enrolled participant via posters only may be more appropriate (CA$300/n = 15 = CA$20/participant). These are similar to direct cost estimates from Adam et al. to a similar nutrition trial: CA$20/participant (social media) and CA$24/participant (offline methods).^18^ To provide a more comprehensive cost evaluation, we have calculated a post-hoc estimation of labour costs in each group: CA$2120 (offline methods) and CA$500 (social media); see breakdown in Supplemental Table 3. Using these estimates, total cost for recruitment via offline methods and social media was CA$2420 ($2120 + $300) and CA$1175 ($500 + $675), and cost per participant was CA$101 ($2420/n = 24) and CA$33 ($1175/n = 36), respectively. Overall, offline methods were much more labour intensive than social media recruitment; these differences are reflected in the total cost.

We provide insight for the optimization of social media advertisements, which is lacking in current literature. Performance of campaigns (clicks, inquiries) consistently improved over time. Numerous factors likely contributed to this. First, automatic optimization was used for all campaigns, which continually improves the advertisement placement and budget allocation. Campaigns may also experience organic growth over time, as the Facebook page receives more ‘likes’ and ‘shares’. The final (‘9th’) campaign was also the longest (18 days), used the maximum budget (CA$100) and used Photo ‘C’, which was the most successful photo as per A/B testing. Facebook Advertising suggests choosing bright, eye-catching, high-resolution photos directly relevant to the brand which convey the message clearly, and avoiding photos with text or small details. Finally, Facebook's default attribution setting was changed from 28-day click, 1-day view to 7-day click, 1-day view in January 2021. This meant that clicks from a shorter period of time were used to inform campaign optimization, thus targeting those who appeared interested (e.g. clicked the advertisement) more frequently. This shorter attribution setting was likely more effective for our campaign durations, which ranged from 10 to 18 days. Overall, clicks, cost per click, inquiries and cost per inquiry were improved by ∼50% in the final campaign.

Although the factors discussed above contributed to optimization of clicks and inquiries, this was not associated with a significant increase in enrolments in social media campaigns. Enrolments per campaign appeared to be more influenced by the time of year. For example, number of enrolees in campaigns ‘3’ (December 2019; n = 1) and ‘6’ (March 2020; n = 0) were notably reduced. This was likely explained by reduced availability of participants around the holidays and onset of the COVID-19 pandemic. Additionally, the highest enrolments occurred in campaign ‘7’ (July 2020; n = 8), which was a time in Vancouver when strict COVID-19 restrictions were lifted, and women may have felt more motivated to leave their homes. Thus, researchers should critically evaluate the time of year when planning social media campaigns.

The lower conversion rate of social media versus offline methods (8% vs. 47%) should not be used to conclude that offline methods were more effective than social media, given that ultimately, social media resulted in a greater number of enrolees, more quickly. However, improving the conversion rate would help to further optimize social media recruitment. A factor likely impacting conversion rate of social media is ease of inquiry. After seeing an online ad, one can immediately message the researcher on the device they are currently using. Additionally, individuals may have clicked on the ad due to initial interest in the photo or campaign aesthetic, without reading the ad details and reflecting on whether they are truly interested in enrolling. Alternatively, inquiring following offline methods requires manually recording contact information and then later inquiring. Thus, these individuals are likely more serious about enrolling at the time of inquiry, presumably explaining the greater conversion rate in this group.

We note various strengths and limitations of the current study. First, we provide numerous tangible strategies for researchers to implement in future online recruitment initiatives. Additionally, this study adds novel evidence which can inform recruitment of a traditionally hard-to-reach population: pregnant women. We had relatively large sample sizes, particularly for analyses of gestational weeks (n = 251), inquiry analyses and calculation of conversion rate (n = 481) and for assessing metrics of online campaigns (see Supplemental Table 2). However, the lack of significance found between groups for education and income may be due to lack of power (as these analyses included only those who enrolled: n = 60), given that differences appear meaningful. The association of campaign budget with clicks and inquiries should be interpreted with caution. Although we found that lifetime budget per campaign was significantly positively associated with clicks and inquiries, this was likely inflated by the final campaign, which had the greatest performance for numerous reasons (e.g. optimization over time, organic growth, attribution setting), in addition to use of the maximum budget of CA$100. Thus, the association of budget on campaign performance (clicks, inquiries) may have been exaggerated, as we could not control for these other factors that improved this campaign's performance. Additionally, it is possible that this small range of dollars spent (CA$50 to CA$100) is not enough to contribute to, or to detect a true difference in, performance metrics by budget.

## Conclusion

Transitioning recruitment methods from offline to online may help researchers to meet recruitment targets more quickly, reducing the labour-intensive burden of traditional offline strategies. Additionally, cost-effectiveness can be improved in future online campaigns by implementing the optimization strategies discussed in this paper. These insights may help to increase clicks and inquiries in future online recruitment initiatives for various study designs. However, this may not be directly correlated to enrolments in clinical research, which appears to depend more on study-specific factors such as time of year and study design (e.g. where study visits are located). Optimization of participant recruitment is vitally important for research success, and ultimately, translation of science into real-world change.

## Supplemental Material

sj-docx-1-dhj-10.1177_20552076221095707 - Supplemental material for Strategies for improving recruitment of pregnant women to clinical research: An evaluation of social media versus traditional offline methods Click here for additional data file. Supplemental material, sj-docx-1-dhj-10.1177_20552076221095707 for Strategies for improving recruitment of pregnant women to clinical research: An evaluation of social media versus traditional offline methods by Kelsey M Cochrane, Jennifer A Hutcheon and Crystal D Karakochuk in Digital Health

sj-docx-2-dhj-10.1177_20552076221095707 - Supplemental material for Strategies for improving recruitment of pregnant women to clinical research: An evaluation of social media versus traditional offline methods Click here for additional data file. Supplemental material, sj-docx-2-dhj-10.1177_20552076221095707 for Strategies for improving recruitment of pregnant women to clinical research: An evaluation of social media versus traditional offline methods by Kelsey M Cochrane, Jennifer A Hutcheon and Crystal D Karakochuk in Digital Health

sj-docx-3-dhj-10.1177_20552076221095707 - Supplemental material for Strategies for improving recruitment of pregnant women to clinical research: An evaluation of social media versus traditional offline methods Click here for additional data file. Supplemental material, sj-docx-3-dhj-10.1177_20552076221095707 for Strategies for improving recruitment of pregnant women to clinical research: An evaluation of social media versus traditional offline methods by Kelsey M Cochrane, Jennifer A Hutcheon and Crystal D Karakochuk in Digital Health
