# Supplementary material for: Strategies for improving recruitment of pregnant women to clinical research: An evaluation of social media versus traditional offline methods
Source: Digit Health. 2022 May 3;8:20552076221095707. doi: 10.1177/20552076221095707 (PMC9069596; doi:10.1177/20552076221095707)
Supplement: sj-docx-1-dhj-10.1177_20552076221095707 - Supplemental material for Strategies for improving recruitment of pregnant women to clinical research: An evaluation of social media versus traditional offline methods [file sj-docx-1-dhj-10.1177_20552076221095707.docx]

**CONSORT 2010 checklist of information to include when reporting a pilot or feasibility randomized trial in a journal or conference abstract**


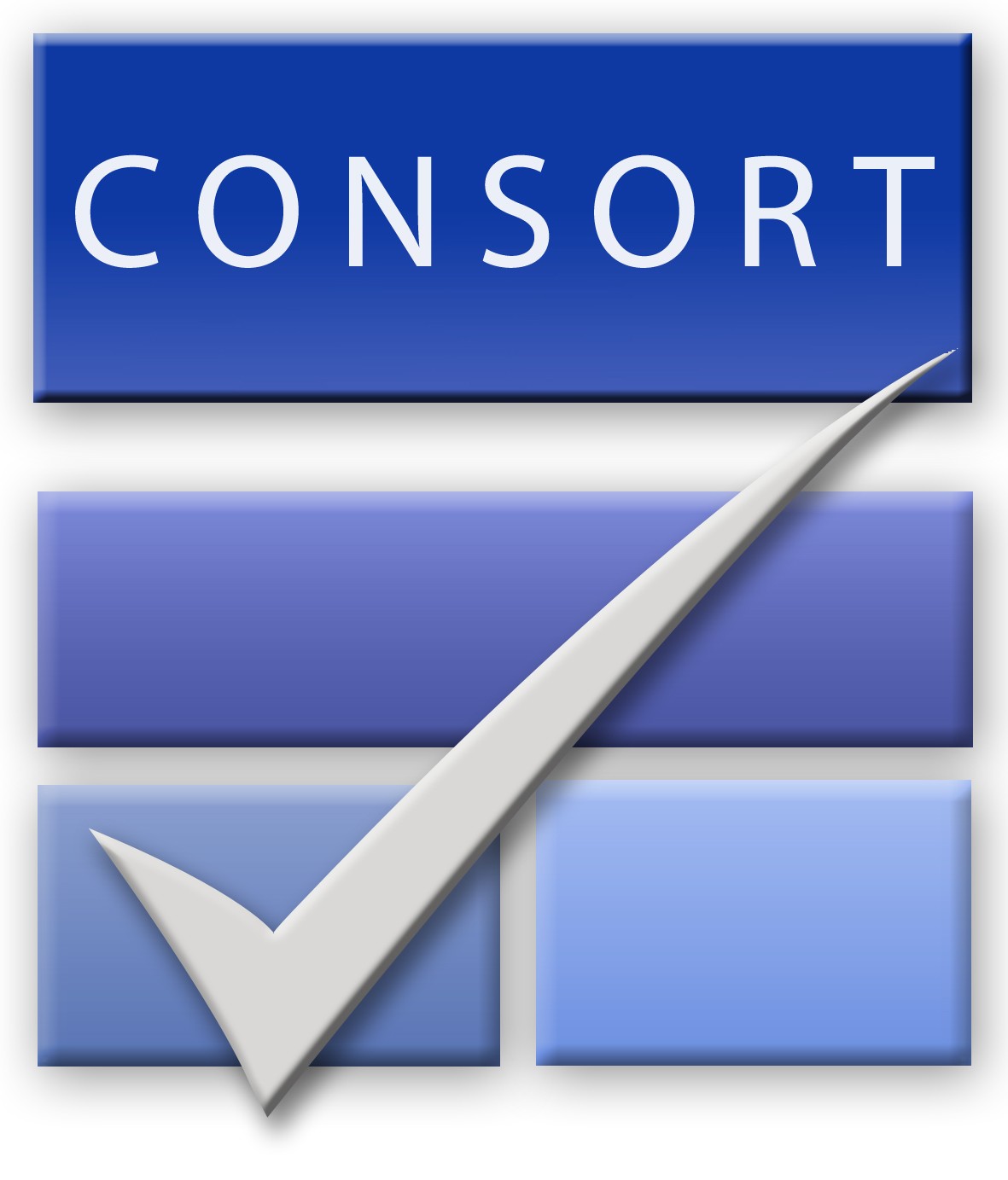


| **Item** | **Description** | **Reported on line number** |
| --- | --- | --- |
| Title | Identification of study as randomised pilot or feasibility trial | Title page (pg 1) |
| Authors * | Contact details for the corresponding author | Title page (pg 1) |
| Trial design | Description of pilot trial design (eg, parallel, cluster) | Lines 67-69 |
| Methods |  |  |
| Participants | Eligibility criteria for participants and the settings where the pilot trial was conducted | Line 71-76  Lines 235-236 |
| Interventions | Interventions intended for each group | Lines 68-69 (but no interventions for the current study; secondary assessment of recruitment) |
| Objective | Specific objectives of the pilot trial | Lines 60-64 |
| Outcome | Prespecified assessment or measurement to address the pilot trial objectives** | See published protocol for full study outcomes (cited on line 67; ref 26)  Lines: 122-144  **Aim 1:**   - Demographic characteristic of those recruit by each method - Gestational weeks - Direct costs - Rate of recruitment - Conversion rate - Odds ratio (odds of enrolment)   **Aim 2:**   - Campaign metrics (reach, impression, clicks, inquires, enrolments) - Association of metrics with dollars spent per campaign - Results of A/B photo testing |
| Randomization | How participants were allocated to interventions | Lines 69-70; See published protocol for full randomization details (cited on line 67; ref 26) |
| Blinding (masking) | Whether or not participants, care givers, and those assessing the outcomes were blinded to group assignment | Lines 69-70; See published protocol for full blinding details (cited on line 67; ref 26) |
| Results |  |  |
| Numbers randomized | Number of participants screened and randomised to each group for the pilot trial objectives** | See published protocol for randomization to each intervention group (cited on line 67; ref 26) |
| Recruitment | Trial status† | Status: Complete |
| Numbers analysed | Number of participants analysed in each group for the pilot objectives** | Lines 146-155  Supplementary Table 2  Figure 2 (CONSORT flow chart) |
| Outcome | Results for the pilot objectives, including any expressions of uncertainty** | Lines 146-194  Lines 318-328 (limitations) |
| Harms | Important adverse events or side effects | N/A  See published protocol for full details (cited on line 67; ref 26) |
| Conclusions | General interpretation of the results of pilot trial and their implications for the future definitive trial | Lines 195-338 |
| Trial registration | Registration number for pilot trial and name of trial register | Title page (pg 1) |
| Funding | Source of funding for pilot trial | Declarations (pg 18) |

Citation: Eldridge SM, Chan CL, Campbell MJ, Bond CM, Hopewell S, Thabane L, et al. CONSORT 2010 statement: extension to randomised pilot and feasibility trials. BMJ. 2016;355.
