# Supplementary material for: Strategies for improving recruitment of pregnant women to clinical research: An evaluation of social media versus traditional offline methods
Source: Digit Health. 2022 May 3;8:20552076221095707. doi: 10.1177/20552076221095707 (PMC9069596; doi:10.1177/20552076221095707)
Supplement: sj-docx-2-dhj-10.1177_20552076221095707 - Supplemental material for Strategies for improving recruitment of pregnant women to clinical research: An evaluation of social media versus traditional offline methods [file sj-docx-2-dhj-10.1177_20552076221095707.docx]

**Supplementary Table 2:** Summary of social media campaign metrics.

| **Campaign** | Total days | Lifetime budget (CAD) | Reach^c^ | Impressions^d^ | Clicks^e^ | Inquiries^f^ | Enrollments | Conversion rate (%)^g^ | Cost per click (CAD)^h^ | Cost per inquiry (CAD)^i^ | Cost per enroll (CAD)^j^ |
| --- | --- | --- | --- | --- | --- | --- | --- | --- | --- | --- | --- |
| **1 (Oct 2019)** | 10 | 100 | 4025 | 7547 | 447 | 49 | 4 | 8 | 0.22 | 2.04 | 25 |
| **2 (Nov 2019)** | 10 | 50 | 2690 | 4730 | 200 | 18 | 5 | 27 | 0.25 | 2.77 | 10 |
| **3 (Dec 2019)** | 10 | 75 | 2900 | 4951 | 261 | 20 | 1 | 5 | 0.29 | 3.75 | 75 |
| **4 (Jan 2020)** | 10 | 50 | 2872 | 5027 | 291 | 29 | 5 | 17 | 0.17 | 1.72 | 10 |
| **5 (Feb 2020)** | 10 | 50 | 3463 | 6130 | 287 | 34 | 2 | 5 | 0.17 | 1.47 | 25 |
| **6 (Mar 2020)** | 15 | 50 | 4476 | 7967 | 300 | 30 | 0 | n/a | 0.17 | 1.66 | n/a |
| **7 (Jul 2020)** | 10 | 100^b^ | 6082 | 10,441 | 594 | 79 | 8 | 10 | 0.17 | 1.27 | 12 |
| **8 (Oct 2020)** | 10 | 100^b^ | 3189 | 6713 | 450 | 35 | 4 | 11 | 0.22 | 2.86 | 25 |
| **9 (Jan 2021)^a^** | 18 | 100 | 5914 | 12,029 | 1190 | 136 | 7 | 5 | 0.08 | 0.74 | 14 |

^a^7-day attribution setting used (all other campaigns used 28-day attribution setting)

^b^Photo A/B testing used: two campaigns run simultaneously, $50/campaign

^c^Reach: number of different people who saw the advertisements

^d^Impressions: number of times the ad was on a screen (may include multiple views by the same person)

^e^Clicks: number of people who clicked on the advertisement

^f^Inquires: number of people who inquired via Facebook messaging or by contacting the research coordinator

^g^Conversion rate: enrollments / inquiries x 100

^h^Cost per click: Lifetime budget (CAD) / clicks

^i^Cost per inquiry: Lifetime budget (CAD) / inquiries

^j^Cost per enroll: Lifetime budget (CAD) / enrollments
