# Supplementary material for: Strategies for improving recruitment of pregnant women to clinical research: An evaluation of social media versus traditional offline methods
Source: Digit Health. 2022 May 3;8:20552076221095707. doi: 10.1177/20552076221095707 (PMC9069596; doi:10.1177/20552076221095707)
Supplement: sj-docx-3-dhj-10.1177_20552076221095707 - Supplemental material for Strategies for improving recruitment of pregnant women to clinical research: An evaluation of social media versus traditional offline methods [file sj-docx-3-dhj-10.1177_20552076221095707.docx]

**Supplementary Table 3:** Breakdown of post-hoc estimation of labour costs.

|  | Recruitment Method | | Cost (CAD)^a^ |
| --- | --- | --- | --- |
|  | Offline | Social Media |  |
|  |  |  |  |
| Poster development & printing | 4 hours | -- | 80 |
| Manual distribution of posters^b^ | 80 hours | -- | 1600 |
| Electronic distribution of posters^c^ | 16 hours | -- | 320 |
| Grand rounds presentation preparation | 4 hours | -- | 80 |
| Presentations (Nov 2019; Sept 2020) | 2 hours | -- | 40 |
| Initial development of online ads | -- | 2 hours | 40 |
| Individual campaign set up^d^ | -- | 5 hours | 100 |
| Responding to inquiries^e^ | -- | 18 hours | 360 |
| **Total hours** | **106 hours** | **25 hours** |  |
| **Total cost (CAD)** | **$2120** | **$500** |  |

^a^Cost estimates are based on $20 CAD/hour compensation

^b^September 2019 to March 2020; estimation of 10 hours/month

^c^April 2020 to December 2020; estimation of 2 hours/month

^d^Approximately 30-minutes of set-up per campaign (x 9 campaigns)

^e^Approximately 10-minutes per active campaign day (x 103 days)
